# Quantifying accuracy and precision from continuous response data in studies of spatial perception and crossmodal recalibration

**DOI:** 10.3758/s13428-024-02416-1

**Published:** 2024-04-29

**Authors:** Patrick Bruns, Caroline Thun, Brigitte Röder

**Affiliations:** Biological Psychology and Neuropsychology, University of Hamburg, Von-Melle-Park 11, 20146 Hamburg, Germany

**Keywords:** Audiovisual, Localization errors, Sensorimotor tasks, Ventriloquism aftereffect, Psychophysics

## Abstract

The ability to detect the absolute location of sensory stimuli can be quantified with either error-based metrics derived from single-trial localization errors or regression-based metrics derived from a linear regression of localization responses on the true stimulus locations. Here we tested the agreement between these two approaches in estimating accuracy and precision in a large sample of 188 subjects who localized auditory stimuli from different azimuthal locations. A subsample of 57 subjects was subsequently exposed to audiovisual stimuli with a consistent spatial disparity before performing the sound localization test again, allowing us to additionally test which of the different metrics best assessed correlations between the amount of crossmodal spatial recalibration and baseline localization performance. First, our findings support a distinction between accuracy and precision. Localization accuracy was mainly reflected in the overall spatial bias and was moderately correlated with precision metrics. However, in our data, the variability of single-trial localization errors (variable error in error-based metrics) and the amount by which the eccentricity of target locations was overestimated (slope in regression-based metrics) were highly correlated, suggesting that intercorrelations between individual metrics need to be carefully considered in spatial perception studies. Secondly, exposure to spatially discrepant audiovisual stimuli resulted in a shift in bias toward the side of the visual stimuli (ventriloquism aftereffect) but did not affect localization precision. The size of the aftereffect shift in bias was at least partly explainable by unspecific test repetition effects, highlighting the need to account for inter-individual baseline differences in studies of spatial learning.

## Introduction

The ability to localize objects and events in space is critically involved in nearly every interaction with the environment and is, thus, vitally important for humans and many other species. Consequently, spatial localization abilities have been a subject of experimental investigations since the dawn of experimental psychology in the nineteenth century (e.g., Stratton, 1897), and numerous psychophysical studies have greatly contributed to identifying the mechanisms underlying spatial perception and learning in humans (for reviews, see Ahveninen et al., 2014; Blauert, 1997; Bruns & Röder, 2019a; Chen & Vroomen, 2013; King, 2009; Middlebrooks & Green, 1991; Recanzone, 2009). A common methodological issue in these studies is the proper quantification of localization performance from continuous response data in sensorimotor tasks. Inauspiciously, different studies have utilized different metrics to quantify localization performance, thereby hampering comparisons between studies. Moreover, the role of inter-individual differences in localization ability for predicting spatial learning outcomes has often been neglected. Therefore, the goal of the present study was to derive recommendations for quantifying localization performance (e.g., in studies of auditory spatial perception and crossmodal recalibration) from a systematic comparison of commonly used metrics.

**Fig. 1:**
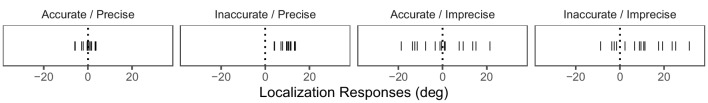
Accuracy and precision in a localization task. *Note.* Panels show simulated data illustrating the theoretical independence of accuracy and precision. Solid lines indicate single-trial localization responses, and the dotted line indicates the actual location of the target stimulus. Localization can be accurate and precise, inaccurate but precise, accurate but imprecise, or neither

On a conceptual level, two aspects of localization performance can be distinguished: accuracy (i.e., the closeness of the average localization response to the actual target location which is also known as spatial bias) and precision (i.e., the average closeness of localization responses to each other). Theoretically, accuracy and precision are independent (Chapanis, 1951; Schmidt et al., 2019): localization performance can be accurate, precise, both, or neither (see Fig. 1). Nevertheless, accuracy and precision could be correlated to varying degrees in actual localization response data because they partially depend on similar factors. For example, studies of auditory localization in the horizontal plane have shown that both accuracy and precision are best for central locations and decrease with increasing eccentricity (Carlile et al., 1997; Makous & Middlebrooks, 1990; Recanzone et al., 1998). In line with these findings, studies in cats (Moore et al., 2008) and humans (Garcia et al., 2017) have suggested a relationship between sensory uncertainty (i.e., precision) and overestimation of peripheral target eccentricity (i.e., accuracy). However, others have observed such a relationship between accuracy and precision only for vertical and not for horizontal sound localization (Ege et al., 2018). Moreover, these studies manipulated sensory uncertainty by changing the signal-to-noise ratio of the sound stimuli in a within-participant design (Ege et al., 2018; Garcia et al., 2017), thus leaving unclear whether the observed changes in accuracy were due to the induced changes in precision or due to changes in the physical properties of the sound stimuli. By contrast, surprisingly few studies have directly assessed the correlation between accuracy and precision metrics across participants.

**Fig. 2:**
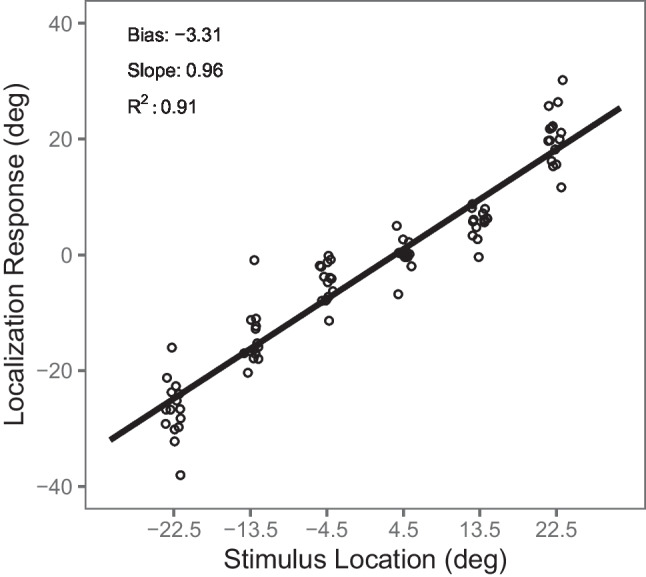
Regression-based localization metrics. *Note.* Single-trial localization responses (*y*-axis) of a randomly selected participant at each azimuthal loudspeaker location (*x*-axis) are indicated by the open circles. The solid line indicates the regression line. Intercept (i.e., bias), slope, and *R*^2^ were taken from the regression model as regression-based localization metrics

In a typical psychophysical task measuring absolute localization abilities, subjects are presented with auditory or visual stimuli from different locations in external space, often restricted to the horizontal plane, and are asked to make pointing, head or eye movements toward the perceived locations of the sources (Bruns et al., 2020b; Ege et al., 2018; Hairston et al., 2003; Lewald, 2002, 2007; Lewald & Ehrenstein, 1998; Ocklenburg et al., 2010; Odegaard et al., 2015; Passamonti et al., 2009; Recanzone, 1998; Recanzone et al., 1998; Strelnikov et al., 2011; Zwiers et al., 2003). Typically, either error-based or regression-based measures of localization performance have been reported in these studies (see Table 1). Error-based measures (see Schmidt et al., 2019) consider the deviation of the localization response from the true target location (i.e., the localization error) in each trial. The mean localization error across trials (usually referred to as constant error or bias) is then construed as an indicator of accuracy and the standard deviation (*SD*) of the single-trial localization errors (usually referred to as variable error) as an indicator of precision (e.g., Bruns et al., 2014; Makous & Middlebrooks, 1990; Recanzone et al., 1998; Odegaard et al., 2015; Perrott et al., 1987). In addition, or as an alternative, the absolute values of the single-trial localization errors are sometimes averaged (usually referred to as absolute error) to yield a composite score of both accuracy and precision (e.g., Bruns et al., 2020b; Makous & Middlebrooks, 1990; Oldfield & Parker, 1984). By contrast, regression-based measures (see Fig. 2) are derived from a linear regression of localization responses on the true stimulus locations. The intercept of the resulting regression line is mathematically equivalent to the constant error or bias and, thus, is an indicator of accuracy. The slope of the regression line captures an over- or underestimation of peripheral locations, which may exist independently of the constant error or bias and which is usually considered a measure of accuracy as well (Ege et al., 2018; Garcia et al., 2017; Lewald, 2007; Ocklenburg et al., 2010). Finally, the coefficient of determination (*R*^2^) has been suggested as a regression-based indicator of localization precision (Lewald, 2007; Ocklenburg et al., 2010).

**Table 1:** Glossary of localization performance metrics

Metric	Derivation	Description
Bias	Error, Regression	Overall bias of localization responses to the left (negative values) or to the right (positive values), equivalent to constant error (CE) and intercept
aCE	Error	Absolute value of bias (or CE), indicates the amount of bias irrespective of direction
maCE	Error	Mean of the aCE per target location, reflects over- or underestimation of peripheral target locations
VE	Error	Mean of the standard deviations (*SD*) of the single-trial localization errors at each target location
pVE	Error	*SD* of the single-trial localization errors pooled across trials from all target locations
AE	Error	Mean of the absolute values of the single-trial localization errors, sensitive to both bias and variability of the localization responses
Slope	Regression	Slope of the regression model function, indicates an overestimation (values > 1) or underestimation (values < 1) of peripheral target locations
*R*^2^	Regression	Coefficient of determination of the regression model, indicates the goodness of the fit of the pointing responses to the regression line

Metrics were derived either from the single-trial localization errors (Error) or from a linear regression of pointing responses on the actual target locations (Regression) for each participant

To clarify the relationship between error-based and regression-based measures of accuracy and precision, the present analysis utilized data from a sound localization task. Auditory localization has received considerable attention in psychophysical studies due to its unique role in spatial perception: sounds can convey spatial information even in complete darkness or for objects outside the visual field. Yet compared to the visual or somatosensory systems, the auditory system is relatively poor in spatial tasks, likely related to the fact that spatial location is not represented directly in the cochlear but has to be inferred from spatial cues generated by the interaction of the sound waves with the head and the external ears (Blauert, 1997; Recanzone & Sutter, 2008). Sound localization in the horizontal plane relies mainly on interaural time differences (ITD) and interaural level differences (ILD) that arise from different arrival times and amplitude levels (due to shadowing of the far ear from the sound source) at the two ears for sound locations deviating from the midline. It is well known that ITDs affect sound localization mainly at frequencies below 1500 Hz due to limitation of phase locking of neurons at high frequencies. By contrast, ILDs are mainly relevant at frequencies above 1500 Hz because low-frequency sounds are bent effectively around the head. This division is known as the duplex theory (for reviews, see Blauert, 1997; King, 2009; Middlebrooks & Green, 1991; Recanzone & Sutter, 2008). Neither ITDs nor ILDs provide information regarding the elevation of a sound source. However, the pinna distorts the frequency spectrum of an incoming sound uniquely depending on its elevation (see also Wightman & Kistler, 1989). Thus, sound localization in the vertical plane relies primarily on these monaural spectral cues, which also allow for some residual capacity to localize sounds in azimuth using one ear alone (Perrott et al., 1987; Van Wanrooij & Van Opstal, 2004).

As a result of the extensive computations underlying auditory spatial perception, errors in sound localization can originate from physical, physiological, and cognitive factors, as well as from the response method used in a study. For example, the eccentricity of peripheral auditory targets is typically overestimated with hand pointing but underestimated with head pointing methods, likely caused by differences in the relative position of the head with respect to the sound source and the trunk inherent in these tasks (Lewald et al., 2000). In addition, the amount of target eccentricity overestimation has been shown to depend on physical properties of the stimuli such as their sound frequency (Blauert, 1997; Lewald & Ehrenstein, 1998), cortical processing of the spatial cues as evidenced in patients with brain lesions (Pinek & Brouchon, 1992), and memory-related processes in delayed-response tasks (Lewald & Ehrenstein, 2001). Thus, identical errors observed in different studies may reflect quite distinct underlying processes which might differently influence localization performance metrics.

Since auditory localization cues change continuously, for example, due to the acoustic properties of the environment, auditory localization additionally requires constant calibration well into adult life (King, 2009; Knudsen, 2002). Studies of crossmodal learning in spatial perception typically compare changes in one or several localization performance metrics before and after an experimental intervention such as exposure to audiovisual stimuli (Bruns et al., 2020b; Strelnikov et al., 2011), light deprivation (Lewald, 2007), or prism adaptation (Zwiers et al., 2003). A particularly fruitful approach has been the study of crossmodal recalibration after experiencing spatially discrepant audiovisual stimuli, commonly referred to as the ventriloquism aftereffect (Bruns, 2019b; Chen & Vroomen, 2013; Recanzone, 2009). After brief exposure to audiovisual stimuli with a consistent spatial disparity (e.g., with the visual stimulus always presented to the right of the auditory stimulus), a change in unimodal auditory constant error or bias is typically observed at the behavioral level (Lewald, 2002; Recanzone, 1998) as well as at the neural level, such that auditory spatial representations in the auditory cortex are shifted toward the side of the visual stimuli (Bruns et al., 2011; Park & Kayser, 2021; Zierul et al., 2017). This visual recalibration of auditory spatial maps is thought to subserve the maintenance of a coherent and accurate multisensory representation of space (Bruns & Röder, 2019a; Recanzone, 2009).

Studies of crossmodal spatial recalibration have typically focused on changes in constant error or bias (i.e., accuracy). Thus, it is unclear whether crossmodal recalibration affects unimodal localization precision as well. Moreover, the amount of crossmodal recalibration (i.e., the size of the ventriloquism aftereffect) might depend on inter-individual differences in localization accuracy and/or precision at baseline. Conceivably, any pre-existing biases of auditory spatial perception in one direction might leave more or less “space” for recalibration depending on the direction of the audiovisual exposure stimuli. In addition, subjects with poor auditory localization precision at pretest might show larger visual recalibration effects than subjects with high baseline localization precision. Such a result would be in line with the well-known role of cue reliabilities in multisensory integration: when estimating the spatial location of an audiovisual stimulus, the visual and auditory cues are typically weighted according to their relative reliabilities (i.e., precision), thereby maximizing the precision with which the audiovisual event can be localized (Alais & Burr, 2004). However, some studies have suggested that crossmodal recalibration might aim at maximizing accuracy rather than precision and, therefore, emerge independently from cue reliability (Rohlf et al., 2021; Zaidel et al., 2011).

To directly compare the different localization performance measures and their ability to predict crossmodal recalibration outcomes at an individual level, we reanalyzed data from two previously published studies (Bruns et al., 2020b; Bruns & Röder, 2019b), resulting in a large sample of 188 healthy adults who had naïvely localized sounds from different azimuthal locations with a pointing stick. In a first step, we calculated both error-based and regression-based localization performance measures for each subject to directly assess the agreement between these two approaches. In a subsample of 57 subjects, data from a second sound localization test, performed after exposure to audiovisual stimuli in which the visual stimulus was consistently presented 13.5° to the right of the sound source, were available. In a second step, we tested in these subjects whether crossmodal recalibration following spatially discrepant audiovisual exposure, which is typically parametrized as a rightward shift in the localization bias (known as the ventriloquism aftereffect), additionally results in changes in any of the other (previously in this setting not tested) sound localization performance measures. This approach allowed us to directly assess the degree to which these measures reflect independent processes. Finally, we tested whether individual performance levels in these measures at baseline were correlated with the observed size of the ventriloquism aftereffect following the audiovisual exposure phase. Based on these analyses, recommendations for quantifying localization performance from continuous response data were derived.

## Method

### Participants

Datasets of 188 healthy adult volunteers (139 women and 49 men; mean age: 24.8 years; age range: 18–46 years) from our previous studies (Bruns et al., 2020b; Bruns & Röder, 2019b), publicly available in the research data repositories of the University of Hamburg (*n* = 120; Bruns et al., 2020a) and the Center for Open Science (*n* = 68; Bruns, 2019a), were reanalyzed for the present study. All participants had provided written informed consent and all experimental procedures had been approved by the ethics commission of the German Psychological Society (DGPs) and were performed in accordance with the ethical standards laid down in the Declaration of Helsinki.

For the present reanalysis, the initial sound localization pretest data for all participants were used (*n* = 188). In addition, data from a sound localization posttest following exposure to audiovisual stimuli with a consistent spatial disparity of 13.5° were analyzed in a subsample of the participants in whom these data were available (*n* = 57). These were the participants in the “LTD fixed incongruent” group (*n* = 15) of Bruns et al. (2020b) as well as the participants in the “constant” group (*n* = 42) of Bruns and Röder (2019b). For the latter group, only the data from the first posttest (of several posttests measured in this study) were considered because these best matched the single posttest data obtained in Bruns et al. (2020b). A sensitivity analysis carried out in G*Power 3.1 (Faul et al., 2009) indicated that the sample size of *n* = 188 had 80% power (at a conventional α level of .05) to detect a correlation of localization performance measures with an effect size of |ρ| = .20. The sample size of *n* = 57 had 80% power (at α = .05) to detect a correlation of pretest sound localization performance and the size of the ventriloquism aftereffect with an effect size of |ρ| = .35.

To control for unspecific test repetition effects, additional control analyses were carried out in a subsample of participants (*n* = 30) who had performed the sound localization test twice but did not receive audiovisual training between tests. These were the “LTD auditory control” and “LTP auditory control” groups of Bruns et al. (2020b).

### Experimental procedure

The experimental procedure has been described in full detail elsewhere (Bruns et al., 2020b; Bruns & Röder, 2019b). Here, we summarize only the procedures and experimental conditions which are relevant for the present reanalysis of the data. In brief, all participants (*n* = 188) naïvely performed a unimodal sound localization test in which they indicated the perceived locations of sounds presented from different azimuthal locations (pretest). A subsample of the participants (*n* = 57) was then exposed to audiovisual stimuli in which the visual component was consistently presented 13.5° to the right of the sound source to induce the ventriloquism aftereffect, and subsequently performed the unimodal sound localization test again (posttest). A separate subsample of the participants (*n* = 30) performed the unimodal sound localization twice but received unimodal auditory stimulation between tests instead.

The experiments were conducted in a 4.70 × 2.35 × 2.25 m soundproof chamber which was treated with sound-absorbing acoustic foam panels (Illbruck, Pinta Acoustic GmbH, Maisach, Germany) and which had an ambient background noise level of 31 dB(A). Auditory stimuli were either 750 Hz tones with a duration of 200 ms (Bruns & Röder, 2019b) or 1000 Hz tones with a duration of 30 ms (Bruns et al., 2020b), both including 5 ms linear rise/fall envelopes and presented at 65 dB(A). Sound intensity was randomly varied over a range of 4 dB for every stimulus presentation to reduce any detectable differences in the loudspeaker transformation functions. Six or eight loudspeaker locations, spanning either ±22.5° (Bruns et al., 2020b) or ±31.5° (Bruns & Röder, 2019b) in steps of 9°, were used. The loudspeakers (ConceptC Satellit, Teufel GmbH, Berlin, Germany) were mounted at ear level on a semicircular frame at a distance of 90 cm and were hidden from view behind an acoustically transparent curtain which extended to ±90° from the participants’ straight-ahead position. A movable red laser pointer was projected onto the curtain for visual stimulation. Participants indicated their responses with a rotatable hand pointer which was mounted in front of them on a crossbar with its pivot in the center of the semicircular frame. The pointer consisted of a metal rod with a length of 30 cm and a diameter of 2 cm. The azimuthal angle of the pointer was recorded from a potentiometer with a resolution of 1° whenever the response button (located on the upper side of the rod approximately 8 cm from the tip) was pressed.

The unimodal sound localization test consisted of 90 or 96 trials, including 15 trials at each of six loudspeaker locations (Bruns et al., 2020b) or 12 trials at each of eight loudspeaker locations (Bruns & Röder, 2019b), which were presented in a randomized order. The red laser point served as a central fixation point at the beginning of each trial. After participants had aligned the hand pointer within ±10° of fixation, the laser point was turned off and the auditory target stimulus was presented with a random delay between 500 and 1500 ms. Participants were instructed to align the hand pointer (using both hands) as accurately as possible with the perceived azimuthal location of the sound source. The next trial started 350 ms after they had confirmed their response with a button press. Responses were not timed except that trials were aborted and counted as a miss if no response was recorded within 10 s from stimulus onset (this occurred in less than 0.1% of trials overall).

In some of the participants, the unimodal sound localization pretest was followed either by an audiovisual exposure block of either 600 trials with a total duration of 300 s (Bruns et al., 2020b) or 200 trials with a total duration of 200 s (Bruns & Röder, 2019b), or by a unimodal auditory exposure block of 200 trials with a total duration of 200 s (Bruns et al., 2020b). In each trial, an auditory stimulus, identical to the stimuli used in the unimodal sound localization test, was presented from one of the loudspeaker locations, either alone (unimodal auditory control condition) or together with a synchronous visual stimulus (red laser point) which was always displaced 13.5° to the right of the sound source (audiovisual recalibration condition). To ensure that participants attended the audiovisual stimulation, they had to detect rare deviant stimuli (i.e., interrupted auditory or visual stimuli or additional visual stimuli) which occurred in 1–4% of the trials, but they did not engage in an active localization task during the audiovisual exposure block. Note that this procedure, as well as the number of audiovisual trials (at least 200), was sufficient to induce maximal ventriloquism aftereffects in previous studies (Frissen et al., 2012). Immediately following the audiovisual exposure block, participants performed the unimodal sound localization test again.

### Data analysis

We calculated both error-based and regression-based localization performance measures (see Table 1) for each participant’s pretest data and, if available, for their posttest data in R version 3.6.2 (analysis code and a working example are available in the UHH Research Data Repository at  10.25592/uhhfdm.10183). To derive *error-based* performance measures (Schmidt et al., 2019), we subtracted the actual auditory target location from the perceived location in each trial. The following metrics were calculated from these single-trial localization errors for each loudspeaker location and then averaged across locations:

- **Bias or constant error (CE):** the mean of the single-trial localization errors. It is equivalent to the intercept in regression-based localization performance measures (see below). The resulting values indicate an overall bias of localization to the left (negative values) or to the right (positive values) of the actual target locations and are, thus, considered a measure of accuracy. It was calculated as

$$CE=\frac{1}{m}\sum\nolimits_{j=1}^{m}\left(\frac{1}{n}\sum\nolimits_{i=1}^{n}\left({x}_{i,j}- {t}_{j}\right)\right)$$

where *n* is the number of trials at each of *m* locations, *x*_*i,j*_ is the localization response in the *i*th trial at the *j*th location, and *t*_*j*_ is the true value of the *j*th location.

- **Absolute constant error (aCE):** the absolute value of the bias or constant error (CE). It is sometimes used to compare the amount of bias (irrespective of direction) between individuals or conditions (e.g., Bruns et al., 2014). It was calculated as   

$$aCE= \left|\frac{1}{m}\sum\nolimits_{j=1}^{m}\left(\frac{1}{n}\sum\nolimits_{i=1}^{n}\left({x}_{i,j}- {t}_{j}\right)\right)\right|$$

- **Mean absolute constant error (maCE):** the mean of the aCE per target location. By averaging the aCE rather than the bias at each location, an over- or underestimation of peripheral target locations is not cancelled out between left and right target locations as in the calculation of bias or aCE. Consequently, the maCE is conceptually related to the slope in regression-based localization performance measures (see below). It was calculated as

$$maCE=\frac{1}{m}\sum\nolimits_{j=1}^{m}\left(\left|\frac{1}{n}\sum\nolimits_{i=1}^{n}\left({x}_{i,j}- {t}_{j}\right)\right|\right)$$

- **Variable error (VE):** the mean of the standard deviations (*SD*) of the single-trial localization errors at each target location. It is an indicator of the variability of the responses and, thus, considered a measure of precision. It was calculated as

$$VE=\frac{1}{m}\sum\nolimits_{j=1}^{m}\sqrt{\frac{\sum\nolimits_{i=1}^{n}{\left({e}_{i,j}- {\overline{e} }_{j}\right)}^{2}}{n-1}}$$

where *e*_*i,j*_ is the signed localization error in the *i*th trial at the *j*th location and ${\overline{e} }_{j}$ is the mean localization error at the *j*th location.

- **Pooled variable error (pVE):** the *SD* of the single-trial localization errors pooled across trials from all target locations. The calculation of the pVE yields non-identical values to the typically reported VE in which *SD* is calculated separately for each target location and then averaged (e.g., Bruns et al., 2014; Garcia et al., 2017; Odegaard et al., 2015). We, therefore, considered both variants of the VE in our analysis. The pVE was calculated as

$$pVE= \sqrt{\frac{\sum_{j,i=1}^{m,n}{\left({e}_{i,j}- \overline{e }\right)}^{2}}{mn-1}}$$

where $\overline{e }$ is the mean localization error across trials from all *m* locations.

- **Absolute error (AE):** the mean of the absolute values of the single-trial localization errors. By disregarding the sign (i.e., direction) of the single-trial errors, the AE is sensitive to both the bias and the variability of the localization responses and, thus, represents a composite measure of both accuracy and precision. It is, therefore, used as a general indicator of localization performance (e.g., Bruns et al., 2020b; Passamonti et al., 2009). It was calculated as

$$AE=\frac{1}{m}\sum\nolimits_{j=1}^{m}\left(\frac{1}{n}\sum\nolimits_{i=1}^{n}\left|{x}_{i,j}- {t}_{j}\right|\right)$$

To derive *regression-based* measures of localization performance, we calculated a simple linear regression of the pointing responses on the actual auditory target locations separately for each participant (see Fig. 2). The following metrics were taken from the linear regression models given by

$${y}_{i,j}= \alpha + \beta {x}_{j}+ {\varepsilon }_{i,j}$$

where *y*_*i,j*_ is the predicted response in the *i*th trial at the *j*th location and *x*_*j*_ is the true value of the *j*th location.

- **Bias or intercept:** the *y*-intercept *α* of the model function. It indicates an overall bias of localization responses to the left (negative values) or to the right (positive values) of the actual target locations and is (assuming equal numbers of trials at each location) mathematically equivalent to the bias or CE in error-based metrics (see above). Thus, in the following, CE and intercept are not reported separately but subsumed under the more general term *bias*.
- **Slope:** the slope *β* of the model function. It indicates an overestimation (values > 1) or underestimation (values < 1) of peripheral target locations that would cancel out in the calculation of the bias. Thus, bias and slope measure different aspects of localization performance, but are usually both considered measures of accuracy (e.g., Lewald, 2007; Ocklenburg et al., 2010). The slope is related (but not equivalent) to the maCE (see above).
- ***R***^**2**^**:** the coefficient of determination of the regression model. It indicates the goodness of the fit of the pointing responses to the regression line and can, thus, be considered a measure of precision (e.g., Lewald, 2007; Ocklenburg et al., 2010). In order to capture the variability of responses, it is crucial that the single-trial localization responses (rather than the mean response per target location) are entered into the regression model.

To quantify the agreement between individual localization performance measures in the pretest data, Pearson correlation coefficients were calculated for each pair of measures. In addition, an exploratory factor analysis (with oblimin rotation) was conducted to identify the underlying factor structure of the data.

In addition, for each metric and each participant with available posttest data (*n* = 57), we calculated the difference between pre- and posttest by subtracting the pretest from the posttest value. Crossmodal recalibration of auditory localization after exposure to spatially misaligned audiovisual stimuli (i.e., the ventriloquism aftereffect) is typically defined as a change in bias (i.e., CE or intercept) from pre- to posttest (Bruns et al., 2020b; Bruns & Röder, 2019b; Lewald, 2002; Recanzone, 1998). Here we tested whether crossmodal recalibration is additionally associated with changes in any of the other sound localization performance measures by comparing for each individual measure the differences between pre- and posttest against zero using one-sample *t* tests. Moreover, we tested whether the individual pretest performance level in any of the sound localization performance measures was correlated with the size of the ventriloquism aftereffect (i.e., the amount of change in bias from pre- to posttest) using Pearson correlation coefficients. As a control for unspecific test repetition effects, we additionally tested the correlation between the change in bias from pre- to posttest and pretest bias in a separate subsample of participants (*n* = 30) who had performed the unimodal sound localization twice but received unimodal auditory stimulation instead of audiovisual stimulation between tests. All statistical tests were additionally performed as Bayesian hypothesis tests in JASP version 0.14 (Wagenmakers et al., 2018) using standard priors, and Bayes factors (BF_10_) are reported.

## Results

### Localization performance measures

**Fig. 3:**
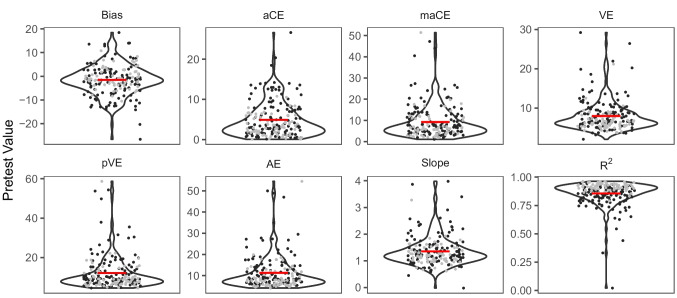
Distributions of individual values in localization performance metrics. *Note.* Single-subject data points are superimposed on violin plots showing the distribution of individual pretest values in each metric. The group mean value is indicated by the red crossbars. The unit of measurement (*y*-axis) is in degrees azimuth except for slope and *R*^2^, which were taken from the regression model. For bias, negative values indicate leftward biases and positive values indicate rightward biases in localization. Dark gray dots indicate the subsample (*n* = 120) from Bruns et al. (2020b), and light gray dots indicate the subsample (*n* = 68) from Bruns and Röder (2019b)

The mean values of the localization performance measures (see Fig. 3) were in a similar range as reported in previous studies of unimodal auditory localization (Bruns et al., 2014; Garcia et al., 2017; Lewald, 2007; Ocklenburg et al., 2010; Odegaard et al., 2015; Oldfield & Parker, 1984). The distribution of individual performances showed, again consistent with previous studies (Odegaard et al., 2015), a considerable amount of heterogeneity in each of the measures (see Fig. 3). On average, participants’ localization responses showed a bias of 1.57° toward the left of the actual auditory target locations, *t*(187) = 3.40, *p* < .001, *d* = 0.25, 95% CI [−2.48, −0.66], BF_10_ = 20.16, consistent with previous observations in predominantly right-handed samples (Ocklenburg et al., 2010; Odegaard et al., 2015). The mean slope of the linear regression model (1.35) was larger than the ideal value of 1, *t*(187) = 8.76, *p* < .001, *d* = 0.64, 95% CI [1.27, 1.43], BF_10_ > 100, indicating that on average participants overestimated the eccentricity of the auditory target locations, which is a well-known finding in hand-pointing tasks (Bruns et al., 2014; Bruns & Röder, 2019b; Garcia et al., 2017; Lewald, 2002; Ocklenburg et al., 2010; Odegaard et al., 2015). Bias and slope (as well as maCE and pVE) did not differ significantly between the subsamples from Bruns et al. (2020b) and Bruns and Röder (2019b), all *p* ≥ .056 (Holm-corrected), all BF_10_ ≤ 1.49, although mean leftward bias (−2.15° vs. −0.55°) and slope (1.42 vs. 1.24) were numerically larger in the subsample from Bruns et al. (2020b) than the subsample from Bruns and Röder (2019b). Significant differences between the two subsamples were observed for the remaining metrics, all *p* ≤ .030, all BF_10_ ≥ 3.49, with larger aCE (5.70° vs. 3.49°), VE (8.69° vs. 6.68°), and AE (12.45° vs. 9.35°) as well as lower *R*^2^ (0.82 vs. 0.91) in the subsample from Bruns et al. (2020b) than in the study by Bruns and Röder (2019b).

**Fig. 4:**
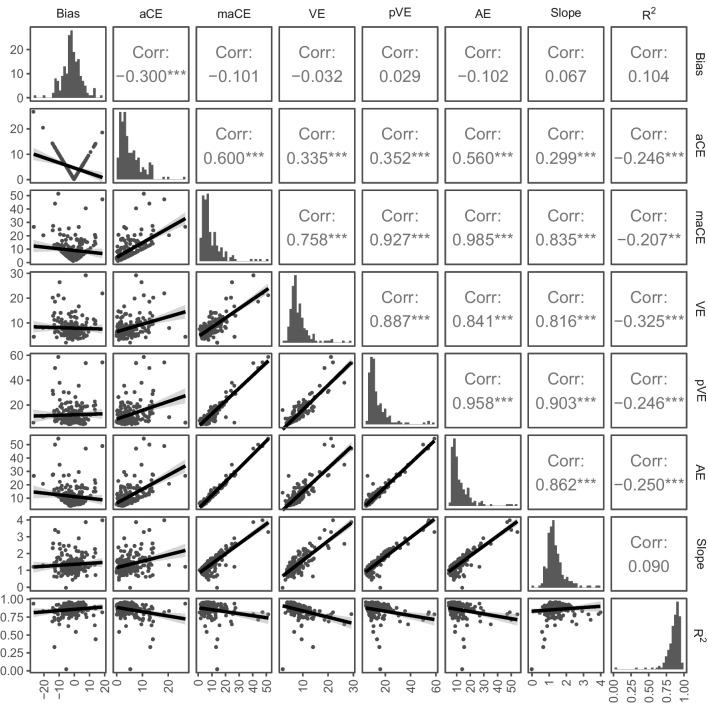
Pairwise correlations of localization performance metrics. *Note.* Scatterplots showing individual data points with regression lines are shown below the diagonal for each pair of metrics. Corresponding Pearson correlation coefficients are indicated above the diagonal. Histograms showing the distribution of individual values in each metric are depicted on the diagonal. The unit of measurement is in degrees azimuth except for slope and *R*^2^, which were taken from the regression model. For bias, negative values indicate leftward biases and positive values indicate rightward biases in localization. *p < .05. **p < .01. ***p < .001

To assess the amount of agreement between the different localization metrics, Pearson correlation coefficients were calculated for each pair of metrics (see Fig. 4). Across the two studies most metrics were significantly correlated except for bias, which was significantly correlated only with aCE (*p* < .001) but with none of the other metrics (all *p* ≥ .157). Bias and aCE are directly related: whereas bias can take both negative and positive values and indicates both the direction and the amount of localization error, aCE (the absolute value of bias) indicates the amount of localization error irrespective of direction. Thus, the negative correlation of bias and aCE (*r* = −.30) simply reflects that the majority of participants showed a leftward bias in localization. The absence of other significant correlations with bias suggests that the direction of bias a subject exhibits is not systematically related to higher or lower levels of accuracy and precision. By contrast, the absolute amount of bias captured by aCE was strongly correlated with maCE (the mean of the aCE per loudspeaker location), *r* = .60, and AE (which is considered a composite score of accuracy and precision), *r* = .56, but less so with slope, *r* = .30, and the precision metrics VE and pVE, *r* ≤ .35, as well as *R*^2^, *r* = −.25. Notably, *R*^2^ was relatively distinct from all other metrics (all |*r*| ≤ .33), whereas the remaining five metrics, VE, pVE, AE, maCE, and slope, showed very strong intercorrelations (all *r* ≥ .76). Thus, measures of over-/underestimation of peripheral locations (maCE, slope), which are typically considered measures of localization accuracy, and measures of localization precision (VE, pVE) were strongly related in our data. Because differences in mean performance were observed with significantly larger errors in the subsample from Bruns et al. (2020b) than in the subsample from Bruns and Röder (2019b) in some of the metrics, we additionally calculated the intercorrelations between metrics separately within each subsample. The resulting pattern was similar in the two subsamples (see Appendix Table 3), suggesting that the intercorrelations between metrics were robust to differences in stimulus duration, sound frequency, and number of locations between the two studies, and collapsing data across the two studies was justified for the purpose of the present analysis.

We used exploratory factor analysis with oblimin rotation to identify the underlying factor structure of our data. According to the Kaiser–Meyer–Olkin measure of sampling adequacy, .70, and Bartlett’s test of sphericity, χ^2^(28) = 2403.32, *p* < .001, factorability of the eight localization metrics could be assumed. Parallel analysis suggested that two factors should be retained, in line with theoretical considerations assuming two latent factors (accuracy and precision). The factor loading matrix for this final solution is presented in Table 2. Overall, measures of over-/underestimation of peripheral locations (maCE, slope), which are typically considered measures of localization accuracy, and measures of localization precision (VE, pVE, AE) loaded on a single factor with high primary loadings above .84. Bias and aCE (which are considered measures of accuracy) as well as *R*^2^ (which is considered a measure of precision) loaded on the second factor, although bias and *R*^2^ were relatively distinct from other metrics (uniqueness above .83), consistent with the correlational analysis reported above.

**Table 2:** Results from a factor analysis of localization performance metrics

Metric	Factor 1	Factor 2	Uniqueness
Bias	0.12	**−0.43**	0.83
aCE	0.23	**0.64**	0.45
maCE	**0.85**	0.27	0.06
VE	**0.84**	0.05	0.26
pVE	**1.00**	−0.03	0.02
AE	**0.90**	0.25	−0.01
Slope	**1.03**	−0.28	0.04
*R*^2^	−0.07	**−0.36**	0.85

The extraction method was principal axis factoring with an oblique (oblimin) rotation. Factor loadings above |0.30| are in bold

#### Crossmodal spatial recalibration

**Fig. 5:**
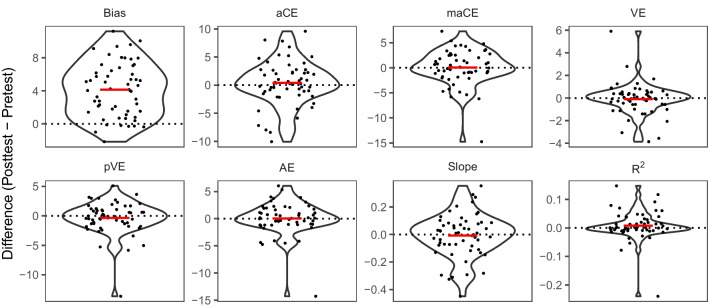
Changes in sound localization performance metrics after audiovisual exposure. *Note.* Performance changes in each metric were calculated by subtracting individual pretest from posttest values. Single-subject data points are superimposed on violin plots showing the distribution of individual difference values in each metric relative to the pretest baseline (indicated by the dotted lines). The group mean value is indicated by the red crossbars. The unit of measurement (*y*-axis) is in degrees azimuth except for slope and *R*^2^, which were taken from the regression model

For participants with available posttest data after exposure to audiovisual stimuli with a constant spatial disparity of 13.5° (*n* = 57), changes in each localization performance metric were calculated as post- minus pretest differences (see Fig. 5). As expected, there was a highly significant rightward shift in bias (*M* = 4.14°) from pre- to posttest, *t*(56) = 9.68, *p* < .001, *d* = 1.28, 95% CI [3.29, 5.00], BF_10_ > 100, which corresponds to the well-known ventriloquism aftereffect (Lewald, 2002; Recanzone, 1998). However, no changes were observed in any of the other localization performance metrics, *p* ≥ .263, *d* ≤ 0.15, BF_10_ ≤ 0.27, suggesting a specific effect of audiovisual exposure on bias.

**Fig. 6:**
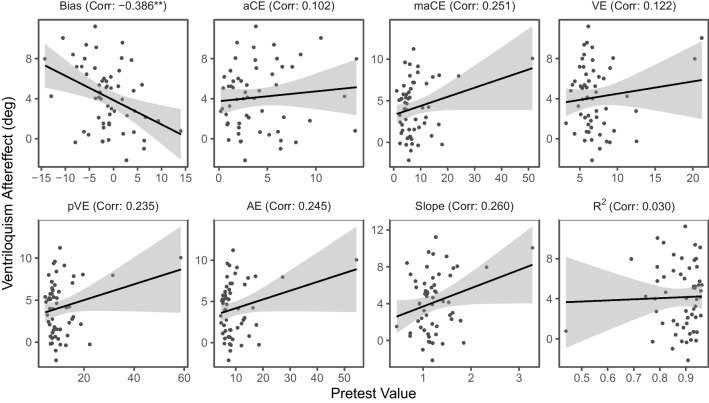
Correlations between pretest performance and size of the ventriloquism aftereffect. *Note.* The size of the ventriloquism aftereffect was calculated by subtracting pretest bias from posttest bias. Scatterplots show individual pretest values in each localization performance metric (*x*-axis) plotted against the ventriloquism aftereffect (*y*-axis) with regression lines. Corresponding Pearson correlation coefficients are indicated in the subplot titles. The unit of measurement is in degrees azimuth except for slope and *R*^2^, which were taken from the regression model. For pretest bias, negative values indicate leftward biases and positive values indicate rightward biases in localization. *p < .05. **p < .01. ***p < .001

We next examined whether the amount of change in bias at posttest (i.e., the size of the ventriloquism aftereffect) was correlated with baseline sound localization performance in any of the metrics at pretest (see Fig. 6). There was a significant negative correlation between the size of the ventriloquism aftereffect and the bias at pretest, *r* = −.39, *p* = .003, 95% CI [−.59, −.14], BF_10_ = 12.12, indicating that stronger leftward biases at baseline were associated with larger ventriloquism aftereffects (i.e., rightward shifts in bias) at posttest. No significant correlations between the size of the ventriloquism aftereffect and any of the remaining sound localization performance metrics at pretest were obtained, *r* ≤ .26, *p* ≥ .050, BF_10_ ≤ 1.07. The significant correlation between baseline bias and ventriloquism aftereffect was mainly accounted for by unspecific test repetition effects, as a similar-sized (but non-significant) correlation, *r* = −.36, *p* = .054, was also observed in a subsample (*n* = 30) that had received unimodal auditory exposure instead of spatially disparate audiovisual exposure between auditory localization tests as a control condition.

## Discussion

In studies of spatial perception including those in a multisensory context, a large variety of different localization performance measures have been used, which can be divided into error-based and regression-based metrics (see Table 1). Yet it has been unknown how these two approaches are interrelated and, thus, no generally accepted guidelines for their usage exist, with different studies reporting different subsets of the available metrics. Here we used a large dataset from 188 individuals who were tested in a sound localization task to directly assess the agreement between error-based and regression-based approaches. Our findings support the theoretical distinction between accuracy and precision and validate the use of (absolute) bias, which can be derived from both error-based and regression-based approaches, as an indicator of localization accuracy. However, our findings additionally show that accuracy and precision metrics can become highly correlated in typical experimental datasets presumably due to common underlying sources of errors. For example, measures of over-/underestimation of peripheral locations (maCE, slope), which are typically considered measures of localization accuracy, and measures of localization precision (VE, pVE) were highly correlated in our data, and we observed a moderate but significant empirical correlation (*r* = .34) between spatial bias (as indexed by aCE) and precision (as indexed by VE) in sound localization. Second, the present results verify that exposure to audiovisual stimuli with a consistent spatial disparity results in a selective shift in bias toward the side of the visual stimuli (the well-known ventriloquism aftereffect), but does not affect localization precision or other aspects of sound localization performance. The size of the ventriloquism aftereffect was dependent on the direction and amount of pre-existing individual localization biases at pretest, but unrelated to baseline performance levels in other metrics. In the following, we will discuss recommendations for quantifying localization performance and implications for crossmodal recalibration studies that arise from these findings. We hope that these recommendations, which are based on findings from a specific experimental setup (Bruns et al., 2020b; Bruns & Röder, 2019b), might serve as a starting point for the development of more generally accepted guidelines for quantifying localization performance and will inspire more extensive studies or meta-analyses involving different experimental designs and tasks.

### Recommendations for quantifying localization performance

Consistent with the conceptual differentiation of localization accuracy and precision, our findings suggest that localization performance metrics can be reduced to two underlying factors. Bias (and its absolute value, aCE), which can be derived from both error-based and regression-based approaches, constitutes the most frequently used metric of localization accuracy (Bruns et al., 2014; Bruns & Röder, 2019b; Lewald, 2002, 2007; Makous & Middlebrooks, 1990; Ocklenburg et al., 2010; Odegaard et al., 2015; Oldfield & Parker, 1984; Perrott et al., 1987; Recanzone et al., 1998) and mainly accounted for one of the two factors. Precision is usually conceptualized as the *SD* of the single-trial localization errors (VE, pVE) in error-based approaches (Bruns et al., 2014; Ocklenburg et al., 2010; Odegaard et al., 2015; Perrott et al., 1987; Recanzone et al., 1998). Our results demonstrate that measures of over-/underestimation of peripheral locations (maCE, slope) can be highly correlated with localization precision (as measured with VE or pVE) and loaded on the same factor in our study, despite being typically considered to reflect localization accuracy rather than precision (Lewald, 2007; Ocklenburg et al., 2010; but see Garcia et al., 2017). Thus, although it may be desirable to directly quantify the amount of over- or underestimation of peripheral targets from a conceptual point of view, researchers need to be aware that common sources of localization errors might exist in their data that could result in largely equivalent values of (p)VE and slope/maCE. Thus, we recommend to explicitly test for this possibility.

Interestingly, *R*^2^ which has been interpreted as a measure of localization precision in regression-based approaches (Lewald, 2007; Ocklenburg et al., 2010), was relatively distinct from all other measures, suggesting that *R*^2^ might be more suitable for quantifying localization precision in situations in which (p)VE and target over-/underestimation are highly correlated due to common underlying error sources. For example, underestimation of target eccentricity has been linked to central tendency biases (Huttenlocher et al., 2000; Odegaard et al., 2015), in which participants would integrate the sensory information (reflecting the actual sensory precision) with the central stimulus location (Aston et al., 2022). Higher weighting of the central location value would result in both stronger underestimation of target eccentricity and lower variability of single-trial responses. It has recently been suggested to correct for central tendency biases by regressing continuous responses on target locations and dividing the variance of the residuals by the squared slope of the regression line (Aston et al., 2022), suggesting that regression-based approaches might be advantageous for estimating localization precision under certain conditions.

In our data, which were derived from a hand-pointing auditory localization task, subjects overestimated the eccentricity of peripheral target locations. The observed high correlation of (p)VE and slope indicates that a larger overestimation of peripheral targets, as reflected in a larger deviation of slope from the ideal value of 1, was tightly linked to larger variability in single-trial localization responses in our data. A similar dependence was reported in a study that manipulated the reliability of auditory stimuli (by varying the level of background noise) within participants (Garcia et al., 2017): in accord with our results, subjects in this study overestimated the eccentricity of less reliable auditory stimuli to a larger extent compared to more reliable auditory stimuli. It remains to be determined whether this relationship similarly holds for the underestimation of peripheral targets, as typically observed in head-pointing tasks (Lewald, 2007; Ocklenburg et al., 2010; Recanzone, 1998), and whether conditions exist in which (p)VE and slope decorrelate.

Although studies of spatial abilities typically report separate measures for accuracy and precision (Bruns et al., 2014; Lewald, 2007; Ocklenburg et al., 2010; Odegaard et al., 2015), relatively few studies have examined the correlation of accuracy and precision (Garcia et al., 2017; Moore et al., 2008). In a large sample of 188 subjects, the present results show a moderate but significant correlation (*r* =.34) between sound localization accuracy and precision metrics, suggesting that although higher accuracy is linked to higher precision empirically, the explained variance of around 12% is relatively small, highlighting the need to estimate both accuracy and precision independently in each subject. In error-based approaches, AE has been used as a composite score of accuracy and precision (Bruns et al., 2014, 2020b; Passamonti et al., 2009). In our data, AE was indeed significantly correlated with both accuracy (aCE) and precision (VE), but this correlation was stronger for precision than for accuracy, suggesting that two dimensions are indeed necessary to fully describe localization performance.

In summary, we suggest the following recommendations for quantifying localization performance from continuous response data:

- All localization performance metrics used in a study should be defined precisely and unambiguously, for example, using the terminology introduced in the present paper (see Table 1).
- Separate metrics should be reported for localization accuracy and localization precision.
- Ideally, both error-based and regression-based metrics should be reported, including at least *bias* (as a standard accuracy metric), *slope* (as an indicator of target eccentricity over-/underestimation), *(p)VE* (as a standard precision metric), and *R*^2^ (as an alternate precision metric).
- In addition, we recommend reporting intercorrelations between metrics, which helps disambiguating localization precision versus target eccentricity over-/underestimation as one aspect of localization accuracy.

In some cases (e.g., in studies comparing different groups or testing interventions in a pretest/posttest design), researchers might wish to focus the statistical analyses on the metric that is a priori considered as best suited to indicate the effect of interest to avoid multiple testing issues. According to our findings, *bias* is relatively distinct from other metrics and might be particularly well suited in studies that focus primarily on localization accuracy. By contrast, in cases in which the primary metric of interest is *slope* (as an indicator of target eccentricity over-/underestimation) or *(p)VE* (as an indicator of precision), it seems advisable to at least exploratorily check the interdependence of the obtained results with other metrics to identify any common and potentially confounding underlying error sources as the ones observed in our study.

### Implications for crossmodal recalibration studies

Crossmodal recalibration (i.e., exposure to spatially discrepant audiovisual stimuli) had a highly selective effect on sound localization accuracy (i.e., bias) in the present sample. This finding confirms that the shift in bias, known as the ventriloquism aftereffect, is indeed due to a crossmodal adjustment of auditory spatial representation to correct for the spatial mismatch (Bruns et al., 2011; Lewald, 2002, Recanzone, 1998; Zierul et al., 2017), rather than due to a higher-order learning of the visual locations (Vroomen & Stekelenburg, 2021). If participants had simply learned the visual locations and used them for localizing the sounds in the posttest, an increase in localization precision (i.e., a reduced VE) would have been expected in addition to the shift in bias due to the usually much higher localization reliability of the visual as compared to the auditory system (Alais & Burr, 2004).

Moreover, our results suggest that the amount of crossmodal recalibration (i.e., the size of the shift in bias) is not significantly affected by individual localization precision at baseline. This is in conflict with the assumption that the amount of crossmodal recalibration is determined by the relative reliabilities of the crossmodal cues presented during adaptation (Burge et al., 2010). It is well known that multisensory integration, as in the ventriloquist situation with spatially discrepant audiovisual stimuli, depends on relative reliability and results in an increase in the precision of the audiovisual estimate (Alais & Burr, 2004; Meijer et al., 2019; Rohlf et al., 2020). It has been argued, however, that crossmodal recalibration aims at maximizing accuracy rather than precision and, thus, might be independent of cue reliability (Zaidel et al., 2011). Accordingly, in a recent study which tested both multisensory integration (ventriloquism effect) and crossmodal recalibration (ventriloquism aftereffect) in the same participants, relative cue reliability (manipulated by blurring the visual stimuli) affected only integration and not recalibration (Rohlf et al., 2021). The present findings add to this that not only short-term manipulations of cue reliability, but also more stable inter-individual differences in localization precision are uninfluential in crossmodal recalibration. On a cautionary note, unimodal visual localization precision at baseline (and hence relative reliability) was not tested directly in the present data. However, in previous studies using the same experimental setup, we found that unimodal visual localization precision was higher and varied far less between participants than auditory localization precision (Bruns et al., 2014; Kramer et al., 2020; Tong et al., 2020). This suggests that unimodal auditory localization precision was a valid proxy for relative cue reliability at baseline, although our analysis might have slightly underestimated the influence of individual localization precision on crossmodal recalibration.

Consistent with a primary aim of crossmodal recalibration to maximize accuracy, we found that the size of the ventriloquism aftereffect shift in bias was best predicted by individual baseline biases. Subjects with strong leftward biases at pretest showed stronger aftereffect shifts in bias toward the right (the side of the visual stimuli during adaptation) than subjects with no biases or rightward biases at pretest. In the audiovisual learning phase of the experiment, no feedback about the veridical location of the stimuli was available. In this situation, subjects seem to put a fixed high weight on the usually more accurate visual input which is not influenced by current visual cue reliability (Rohlf et al., 2021; Zaidel et al., 2011) but may rather be acquired (Rohlf et al., 2020), possibly during a sensitive period in development (Badde, Ley et al., 2020a; King, 2009). Thus, pre-existing individual biases could in principle determine the amount of adjustment that is necessary to correct for the audiovisual spatial mismatch and account in part for the inter-individual variability in the size of the ventriloquism aftereffect.

The observed dependence of crossmodal spatial recalibration on pre-existing spatial biases is consistent with studies of audiovisual temporal processing (Grabot & Kayser, 2020; Stevenson et al., 2012). Whereas inter-individual differences in the size of the temporal binding window, which reflect the precision of audiovisual temporal perception, were linked to the amount of multisensory integration in the McGurk and sound-induced flash illusions (Stevenson et al., 2012), inter-individual differences in temporal biases (reflecting accuracy) were related to crossmodal temporal recalibration processes (Grabot & Kayser, 2020). Inter-individual differences in perceptual biases were found to be highly stable across time (Badde, Ley et al., 2020a; Grabot & van Wassenhove, 2017; Odegaard & Shams, 2016) and, thus, their interaction with short-term experimental manipulations needs to be taken into account. However, results from our control analysis in participants who had performed the sound localization test twice but without interjacent spatially discrepant audiovisual exposure suggested that an apparent influence of baseline biases on crossmodal recalibration might be exaggerated by measurement errors present at baseline. Due to a simple regression toward the mean effect, an artificial correlation between baseline biases and the amount and direction of change in bias from pre- to posttest would necessarily be introduced which might superimpose any underlying correlation in perception.

Thus, in crossmodal recalibration studies in which the primary focus is on isolating the effect of an experimental manipulation in a pre-/posttest design, precautions should be taken to minimize influences of measurement errors on estimated learning outcomes:

- First, rigorous baseline measurements have to be introduced to counteract any measurement errors. This could, for example, be achieved by adding an extensive practice period before the actual measurement (Carlile et al., 1997; Oldfield & Parker, 1984) or by taking repeated baseline measurements until performance converges before introducing the experimental manipulation of interest (Dinse et al., 2006; Godde et al., 2000). Of course, the extent of the baseline measurement needs to be balanced with potentially adverse effects of elongating the duration of the experiment such as participant fatigue, which might be particularly relevant in studies involving children or patient groups.
- Second, for any remaining perceptual biases that are not due to measurement errors, one strategy might be to correct the experimental manipulation accordingly. For example, in a study of the ventriloquism aftereffect, the physical audiovisual spatial disparity could be individually adjusted to equalize the perceived audiovisual spatial disparity between participants: If, for instance, the targeted audiovisual spatial disparity is 10°, a participant with a leftward perceptual bias of 2° at baseline would be presented with an actual audiovisual disparity of 8° whereas a participant with a rightward perceptual bias of 2° would be presented with an actual audiovisual spatial disparity of 12°, so that the perceived spatial disparity would be 10° in both cases. Ideally, such an individual adjustment should take both auditory and visual baseline localization biases into account and would, thus, require an additional visual localization measurement at baseline.

### Generalizability and limitations of the present findings

Our assessment of error-based and regression-based localization performance metrics was based on hand-pointing data from a sound localization task that was restricted to the central region of space (±22.5° to ±31.5°) and that used relatively short stimuli (30 or 200 ms). Thus, the precise intercorrelation values between different metrics that we observed in the present study may not necessarily be generalizable to other experimental designs or sensory modalities. It is a well-known finding that errors in sound localization tasks differ depending on the experimental conditions, stimuli, instructions, and psychophysical methods employed to measure localization performance (Blauert, 1997; Carlile et al., 1997; Lewald et al., 2000; Lewald & Ehrenstein, 2001; Perrott et al., 1987; Pinek & Brouchon, 1992; Recanzone et al., 1998; Wightman & Kistler, 1989).

For example, auditory target eccentricity is typically overestimated with hand-pointing tasks but underestimated with head-pointing tasks, likely due to inherent differences in head position involved in these two tasks (Lewald et al., 2000; Ocklenburg et al., 2010; Pinek & Brouchon, 1992). In addition, the amount of overestimation errors may depend on technical causes such as a slight parallax between the pointer pivot and head position (Lewald et al., 2000), the sound frequency spectrum (and its filtering by the external ear) of the involved stimuli (Lewald & Ehrenstein, 1998), and memory-related processes for short stimulus durations (as the ones used in the present study) that require a response to the remembered sound location rather than to an ongoing sound (Lewald & Ehrenstein, 2001). The presence or absence of these different sources of error may contribute differently to localization precision metrics such as p(VE), thereby modulating any correlation between localization accuracy and precision metrics.

Moreover, the processes involved in sound localization errors, including the physical processes involved in the interaction of sound waves with the pinna, may be a unique characteristic of the auditory system. Thus, to which degree the present results and suggestions are transferable to the visual and somatosensory systems, in which stimulus location is more directly represented at the receptor level, needs further investigation. We speculate that interdependencies between different localization performance metrics likely exist in other sensory modalities and tasks as well. For example, the presence of any central tendency biases would introduce a correlation between localization precision, as measured with the p(VE), and an underestimation of target eccentricity reflected in maCE or slope (Aston et al., 2022). Therefore, we propose that our general recommendations for quantifying localization performance from continuous response data apply to a wider range of tasks including spatial localization tasks in other sensory domains like vision (Lewald, 2002; Odegaard et al., 2015) and touch (Badde, Navarro et al., 2020b; Samad & Shams, 2016) as well as temporal perception tasks (Polti et al., 2018), although the specific interdependencies between error-based and regression-based metrics in these scenarios might be different.

## Conclusions

In summary, localization performance was well defined by the two dimensions of accuracy and precision. Our findings demonstrate that accuracy metrics, in particular those measuring target eccentricity over-/underestimation (maCE, slope), and precision metrics, in particular p(VE), can become highly correlated presumably due to shared underlying sources of error (see also Garcia et al., 2017). Hence, we consider it essential to report an exhaustive set of both error-based and regression-based metrics (ideally including *R*^2^ as an alternative precision metric) and to consider intercorrelations between individual metrics in studies of spatial perception. Moreover, crossmodal recalibration as assessed with the ventriloquism aftereffect resulted in a selective shift in spatial bias which was not influenced by baseline localization precision in our data. Here we found that this shift in spatial bias might at least partly be explainable by unspecific test repetition effects. These results highlight the need to account for inter-individual baseline differences in localization metrics in studies of spatial learning (Grabot & Kayser, 2020). Although the present study focused on auditory spatial perception and crossmodal recalibration of sound localization, similar interdependencies between error-based and regression-based metrics might emerge in other sensory domains (Aston et al., 2022) as well as temporal perception tasks (Polti et al., 2018), and we recommend explicitly testing for this possibility in future research. We hope that our recommendations will motivate the development of more generally accepted guidelines for the usage of localization performance metrics derived from continuous response data.

## Data Availability

This article is based on previously published data (Bruns, 2019a; Bruns et al., 2020a). The data and materials that support the findings of the present study are available in the UHH Research Data Repository at 10.25592/uhhfdm.10183.

## Appendix

**Table 3:** Pairwise correlations of localization performance metrics in each subsample

Metric	1	2	3	4	5	6	7	8
1. Bias	-	−.336^***^	−.063	.054	.123	−.050	.146	.052
2. aCE	.031	-	.630^***^	.289^**^	.330^***^	.570^***^	.278^**^	−.148
3. maCE	−.167	.472^***^	-	.746^***^	.906^***^	.983^***^	.827^***^	−.140
4. VE	−.298^*^	.312^**^	.829^***^	-	.896^***^	.835^***^	.810^***^	−.229^*^
5. pVE	−.235	.350^**^	.978^***^	.899^***^	-	.946^***^	.905^***^	−.188^*^
6. AE	−.204	.446^***^	.992^***^	.876^***^	.991^***^	-	.857^***^	−.172
7. Slope	−.188	.242^*^	.876^***^	.823^***^	.905^***^	.883^***^	-	.205^*^
8. *R*^2^	.147	−.358^**^	−.421^***^	−.548^***^	−.449^***^	−.461^***^	−.107	-

The results for the subsample (*n* = 120) from Bruns et al. (2020b) are shown above the diagonal. The results for the subsample (*n* = 68) from Bruns and Röder (2019b) are shown below the diagonal

^*^*p* < .05. ^**^*p* < .01. ^***^*p* < .001

## Code availability

Analysis code to extract error-based and regression-based localization performance metrics from single-trial response data including a working example is available in the UHH Research Data Repository at 10.25592/uhhfdm.10183.
